# PHOTO-ELICITATION- A VIEW OF DEEP OLD AGE AND SOCIAL INCLUSION

**DOI:** 10.1093/geroni/igac059.2904

**Published:** 2022-12-20

**Authors:** Elaine Eliopoulos

**Affiliations:** University College London, London, England, United Kingdom

## Abstract

Photo-elicitation- A View of Deep Old Age and Social Inclusion While visual methodologies have gained prominence in aging research, focus on deep old age has been uncommon. The variety of visual representations portraying a range of ‘ageless aging’ to frail older people may not fully capture daily lived experience. The inclusion of those in deep old age to depict their aging bodies is a crucial missing element enhancing our understanding of the nuances of deep old age. This study fills that gap. Photo-elicitation, followed by semi-structured interviews, was used to examine the role of the body for participants 80 years+ in three US island contexts to understand what impact, if any, their bodies had on their ability to be socially included in ways they chose. Digital cameras were provided to participants to photograph their lived bodily experience. Those who had visual challenges were assisted in taking photographs. Recent analysis of the photographs revealed important findings that will contribute to theoretical development of deep old age: participants who took photos without assistance did not take a single photograph of their bodies. They took photos primarily of their environment and places of community connections while minimising physical challenges. Their photos contextualised their experience of the physical challenges that played a role in their sense of inclusion within their communities. This finding questions current dominant models of the third and fourth age as dichotomous formulations related to embodied agency. In doing so it opens new possibilities for theoretical reflection on deep old age.

